# The Project Baseline Health Study: a step towards a broader mission to map human health

**DOI:** 10.1038/s41746-020-0290-y

**Published:** 2020-06-05

**Authors:** Kristine Arges, Themistocles Assimes, Vikram Bajaj, Suresh Balu, Mustafa R. Bashir, Laura Beskow, Rosalia Blanco, Robert Califf, Paul Campbell, Larry Carin, Victoria Christian, Scott Cousins, Millie Das, Marie Dockery, Pamela S. Douglas, Ashley Dunham, Julie Eckstrand, Dominik Fleischmann, Emily Ford, Elizabeth Fraulo, John French, Sanjiv S. Gambhir, Geoffrey S. Ginsburg, Robert C. Green, Francois Haddad, Adrian Hernandez, John Hernandez, Erich S. Huang, Glenn Jaffe, Daniel King, Lynne H. Koweek, Curtis Langlotz, Yaping J. Liao, Kenneth W. Mahaffey, Kelly Marcom, William J. Marks, David Maron, Reid McCabe, Shannon McCall, Rebecca McCue, Jessica Mega, David Miller, Lawrence H. Muhlbaier, Rajan Munshi, L. Kristin Newby, Ezra Pak-Harvey, Bray Patrick-Lake, Michael Pencina, Eric D. Peterson, Fatima Rodriguez, Scarlet Shore, Svati Shah, Steven Shipes, George Sledge, Susie Spielman, Ryan Spitler, Terry Schaack, Geeta Swamy, Martin J. Willemink, Charlene A. Wong

**Affiliations:** 1 Duke University, School of Medicine, Durham, NC USA; 2 Stanford University, School of Medicine, Stanford, CA USA; 3 Vanderbilt University, School of Medicine, Nashville, TN USA; 4 Verily Inc., South San Francisco, CA USA; 5 Harvard University, School of Medicine, Boston, MA USA; 6 Google Inc., Mountain View, CA USA; 7 California Health and Longevity Institute, Westlake Village, CA USA

**Keywords:** Biomarkers, Data integration, Cancer, Cardiovascular diseases, Diagnosis

## Abstract

The Project Baseline Health Study (PBHS) was launched to map human health through a comprehensive understanding of both the health of an individual and how it relates to the broader population. The study will contribute to the creation of a biomedical information system that accounts for the highly complex interplay of biological, behavioral, environmental, and social systems. The PBHS is a prospective, multicenter, longitudinal cohort study that aims to enroll thousands of participants with diverse backgrounds who are representative of the entire health spectrum. Enrolled participants will be evaluated serially using clinical, molecular, imaging, sensor, self-reported, behavioral, psychological, environmental, and other health-related measurements. An initial deeply phenotyped cohort will inform the development of a large, expanded virtual cohort. The PBHS will contribute to precision health and medicine by integrating state of the art testing, longitudinal monitoring and participant engagement, and by contributing to the development of an improved platform for data sharing and analysis.

## Introduction

Dramatic advances in digital, molecular, and imaging technology used in both research and healthcare delivery are leading to pivotal changes in our understanding of health and the transition to disease. Innovations such as miniature sensors are changing the mechanisms we use to collect data and the quantity of data we can collect to better understand the health and illnesses of individuals and populations. People themselves are collecting and reporting more data about their own health and increasingly wish to be involved in decisions about their own health care^1^. Critical interactions among biology, behavior, the environment and social systems have been well documented^2–4^. However, until recently we lacked the storage capacity and computational power to accrue and analyze relevant information because of its vast complexity and scale. As the capacity to integrate multidimensional information advances, researchers and health care organizations will have an empirical evidence base to promote new collaborative research and care paradigms that include family, clinicians, patients, and the public health system.

The PBHS is designed to establish a reference health state and to develop a platform that integrates and analyzes personalized, longitudinal multi-dimensional data, including a more continuous time dimension than in the past. Some of these data can be generated within a traditional clinical context, but much of it will come from the day-to-day life of people outside of conventional medical research or clinical care settings. The analysis of data gathered through this study will allow for previously disparate information to inform both precision (disease prevention and earlier detection based on individual risk)^5^ and population health (the health outcomes of a group of individuals)^6^.

Changes in the cadence of data collection from episodic to continuous, as well as the scale of data collection from gigabytes to terabytes per individual necessitates an updated framework to collect, organize, analyze, and activate comprehensive health information. The project brings together partnerships among academia, the technology industry, non-profit organizations, healthcare delivery systems and, most importantly, people who are both healthy and ill. The study was designed to be adaptive to what is learned and to advancing technology to explore in depth biological variability of healthy individuals or people with chronic disease over time and to establish reference health states that integrate multiple health dimensions.

## Project Baseline Health Study Design

The PBHS has an initial enrollment goal of at least ten thousand participants, beginning with intensive measurement in the first 2,500 [the deeply phenotyped cohort (DPC)] in whom a large volume of multimodality data is collected, evolving to a broad system involving remote and “in person” components including a blend of virtual and face-to-face research activity. Four clinical PBHS sites in the United States have begun enrollment. A pre-Project Baseline pilot was also conducted for 200 healthy participants prior to initiation of the primary study, which tested clinical assessment workflows. At study initiation a virtual registry was created, and this platform is now being extended to a population orders of magnitude larger with less comprehensive data collection for each person. The registry is designed to offer a simple entry point for participants and enable an easier method for screening and enrolling participants with appropriate population characteristics, and to optimize study flow into the DPC of the PBHS or other studies. The PBHS is funded by Verily and managed in collaboration with Stanford and Duke Universities and the California Health and Longevity Institute, while the extended studies have governance approaches specific to the needs of each study. This manuscript focuses on the PBHS and the DPC and discusses the extended Baseline platform to provide perspective on the goals and strategic approaches currently being considered for the overall effort.

## Study objectives

The objectives of the PBHS are: (i) develop a set of scalable and standardized tools and technologies to collect, organize, and analyze clinical, molecular, imaging, sensor, self-reported, behavioral, psychological, environmental, and other health-related measurements; (ii) evaluate the use of sensor technologies for the collection of more continuous, accurate health information; (iii) create a dataset encompassing a wide spectrum of phenotypic measures; (iv) measure the phenotypic diversity observed among a participant population and its trajectory in health and disease; and (v) share data with qualified investigators to extend learning and create an example of open science.

The PBHS is intended to be observational and correlational, laying the groundwork for discovery. The compilation of the acquired information will lead to a dataset encompassing a wide spectrum of molecular and phenotypic measures for exploratory analyses, to measure the phenotypic diversity observed among the participant population, and to define a range of expected values for specific data types. This data collection effort is intended to drive and support an adaptable study design and future hypothesis testing by the biomedical community. Qualified investigators from the global community will be able to access study data through the Verily Terra platform (https://terra.bio/) after an interval deemed by the Executive Committee to be adequate for a multidimensional data set to be ready for analysis, during which the collaborating institutions have access with Project Baseline Executive Committee approval. The Executive Committee and all collaborating institutions are committed to ensuring data access to the larger community and are testing that process through the platform within the collaborative institutions. For wider access, methods and standards will need to provide rigorous protections for dealing with de-identification in an era in which such biological data can be more readily re-identified. This will require evolution of the technical standards for making data available, plus considerations of qualifications of researchers, oversight by ethics organization (IRBs) and obligations required of those who access the data to ensure appropriate dissemination of results from the data.

## Project Baseline Health Registry and Recruitment

**Fig. 1:**
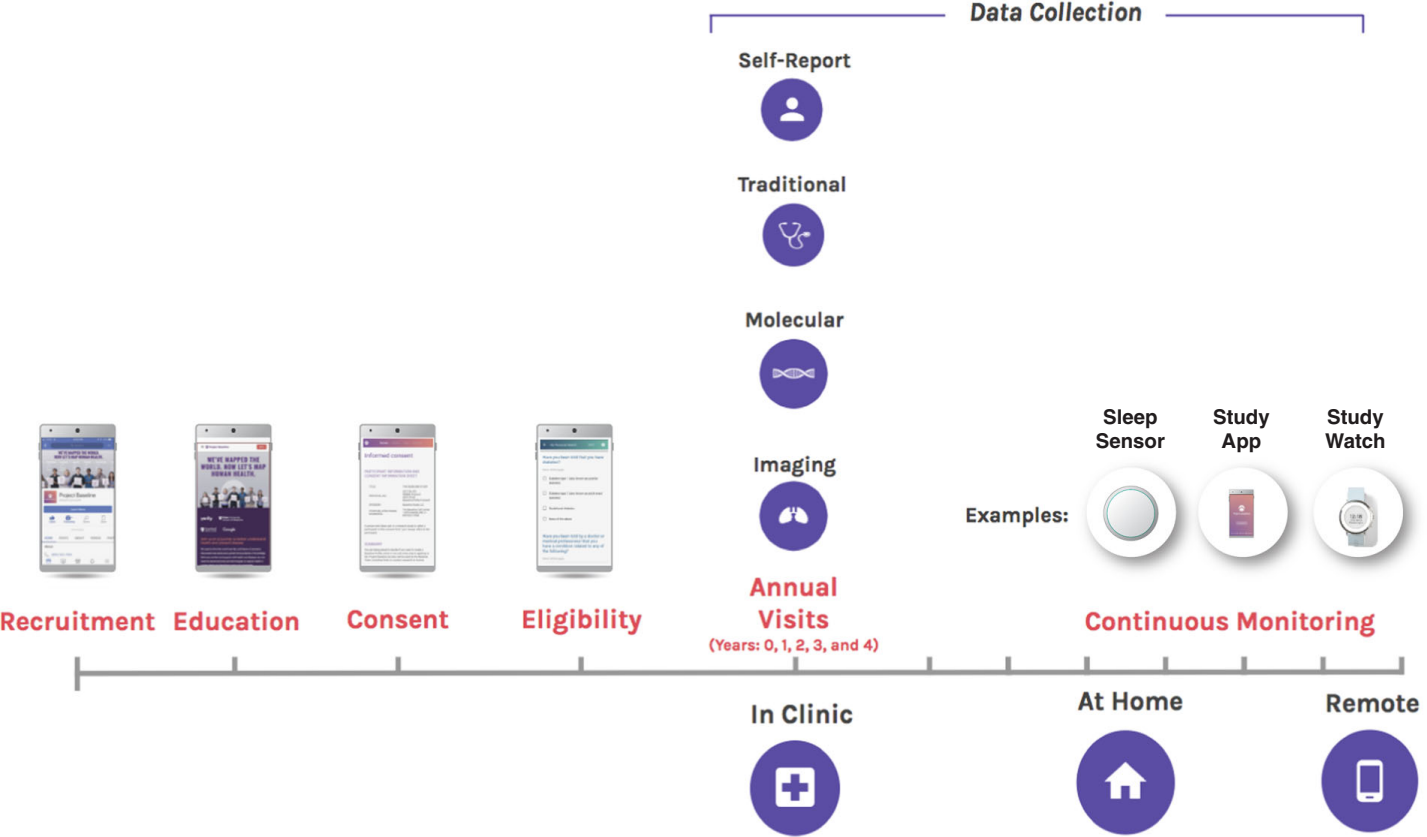
Overview of participant flow. Participants are recruited and screened. After the initial screening period, participants have annual follow-up visits in person. A broad range of health measurements are conducted in clinic, at home, and remotely. Participants are able to provide input and have access to communication with study staff on a more continuous basis between study visits. The current duration of the follow up is to occur over the course of the next four years. ^*^Study watch image used with permission from Verily Inc.

Participants are being recruited primarily through an online registry (Fig. 1). All study components including the registry are currently in English, however materials will be developed in relevant languages as the virtual registry develops. Potential participants are identified through IRB-approved advertisements and clinician recommendations; sites also may refer potential participants based on electronic health medical record review or by proactively recruiting potential volunteers through a variety of community engagement activities. All volunteers are directed to visit the Project Baseline website (www.projectbaseline.com) or to connect with a call center to learn more about the study and enroll in the registry. Selected registrants are invited to join the cohort study based on demographics and disease risk patterns, while the remainder are kept on an active waiting list from which they may have other opportunities to engage in clinical research. Selection of participants is designed to ensure a representative cohort as described below. Written consent is obtained from all participants enrolled in the PBHS and the study is approved by both a central IRB (Western IRB) and IRBs at each of the participating institutions.

### Study population

The study population is selected from the registry to include a broad range of participants across the entire health spectrum, including those who exhibit “exceptional” health (by known standards), varying levels of disease risk, and those already with a disease diagnosis. The initial deeply phenotyped population is enriched (as described in the study design section of the Supplementary Information and Supplementary Figs. 1 and 2) to have an ~60% higher risk relative to participants of the same age and sex for breast/ovarian cancer, lung cancer, and/or atherosclerotic cardiovascular disease (CVD), in approximately equal proportions.

CVD and cancer are selected for enrichment because they are the leading causes of death in the U.S. and globally^7^ and because a sufficient body of literature suggests the possibility of identifying unique combinations of measures and/or biomarkers that could lead to subsequent studies of interventions. CVD is the leading cause of death for both men and women; 610,000 deaths occur each year, constituting one in every four deaths in the U.S.^8^. One in eight American women (~12%) will develop invasive breast cancer over the course of her lifetime. In 2016, there were more than 2.8 million women with a history of breast cancer in the U.S., including women currently being treated and women who had finished treatment^9^. Ovarian cancer affects 20,000 American women a year, with 14,000 related deaths^10^. In 2016, there were 224,390 new cases of lung cancer detected with 155,000 related deaths^11^. Importantly, each of these diseases has a significant prevalence within the U.S. population and a significant body of literature and clinical understanding that may be used for actionable guidance. Clear evidence exists that patients with breast/ovarian^12,13^ and lung^14^ cancers and CVD^15^ benefit from early detection and diagnosis with improved outcomes achieved through known interventions. As other areas of interest develop, the PBHS is designed to be adaptable to enable enrollment of specific new populations and disease conditions.

To achieve broad impact, the aggregate demographic and clinical characteristics of participants are actively monitored to ensure that the study population reflects a diverse racial and ethnic distribution similar to the U.S. census data and adheres to the continuum of health and disease states expected in the research participant population. For the initial portion of the study the minority of interested people were enrolled in the DPC, but all were included in the online registry (Fig. 1). The initial enrolled population has been stratified by age and sex to achieve a representative population with regard to these characteristics. Selected baseline demographic, virtal sign and laboratory characteristics of the first 2502 participants are shown in Table 1.

**Table 1:** Demographics of the initial participants.

	Palo Alto (*N* = 1009)		Durham (*N* = 485)		Kannapolis (*N* = 492)		LA (*N* = 516)		All BHS sites (*N* = 2502)	
Variable	*n*	Mean (%)	*n*	Mean (%)	*n*	Mean (%)	*n*	Mean (%)	*n*	Mean (%)
Age <45	404	40.0	183	37.7	290	58.9	154	29.8	1031	41.2
Age 45–64	285	28.2	228	47.0	162	32.9	212	41.1	887	35.5
Age 65+	320	31.7	74	15.3	40	8.1	150	29.1	584	23.3
Female	529	52.4	288	59.4	260	52.8	298	57.8	1375	55.0
Male	480	47.6	197	40.6	232	47.2	218	42.2	1127	45.0
White	650	64.4	224	46.2	324	65.9	384	74.4	1582	63.2
Black or African American	72	7.1	194	40.0	42	8.5	92	17.8	400	16.0
Asian	165	16.4	35	7.2	54	11.0	6	1.2	260	10.4
American Indian or Native American	10	1.0	8	1.6	6	1.2	7	1.4	31	1.2
Hawaiian or Pacific Islander	13	1.3	4	0.8	10	2.0	0	0.0	27	1.1
Other	99	9.8	20	4.1	56	11.4	27	5.2	202	8.1
Hispanic	140	13.9	20	4.1	79	16.1	52	10.1	291	11.6

Demographics summary by site for the first 2502 participants.

The Duke study sites consist of both Durham and Kannapolis.

### Study schedule and follow-up visits

Participant enrollment and data collection for the Project Baseline Health Study began in 2017. The deeply phenotyped participants will be followed for at least four years, after which decisions about the depth of data collected in further follow-up will be made based on learning from the study. Participants will attend annual follow-up visits, complete quarterly questionnaires, and be monitored through sensors and other participant-centered technology. At each annual visit, a series of study assessments will be conducted, as described below. Participants will be encouraged to notify site personnel throughout the study of changes in their health status or sense of wellbeing and to report all medical encounters (e.g., clinic, urgent care, emergency department visits, or hospitalizations) primarily through the 24-h participant web portal and mobile application. Participants are periodically re-contacted for completion of protocol mandated procedures and intensive efforts to improve the evaluation of technologies. If needed, participants can receive support from staff at any point during the study.

### Data collection and assessments

Detailed assessments are collected at study visits to include a broad and deep array of measurements as detailed in Table 2. The assessments were selected based on the potential for scientific yield, the time to perform the assessment, reproducibility, risk to participant, and cost. The choice of sensors was based on ease of use, likely engagement by participants, reproducibility, and information yield. Additional assessments and sensors are being added or subtracted using an iterative approach as the study databases evolve and the analyses are performed. Access to EHR data has been consented, so that more detailed historical, laboratory, and imaging data are available from prior to enrollment and in follow-up. Population-based aggregate and environmental data such as local and national census data, socioeconomic data, and Centers for Medicare & Medicaid Services (CMS) data may also be included using evolving methods such as Blue Button integration^16^. Additional datasets, including third-party data, may be included in the integrated study database. Further, while some participants’ samples have been assayed with a broad array of tests (Tables 2–4), participant samples will be stored for in depth testing at a later time when current assays are pertinent or new assays are available or new understanding of a disease process make performing a standard assay, not originally done, to be done on all or a sub-group of participants because of this new knowledge.

**Table 2:** Study data types.

	Study start visit	Quarterly follow-ups^a^	Annual visit	Quarterly follow-ups^a^	Annual visit	Quarterly follow-ups^a^	Annual visit	Quarterly follow-ups^a^	Annual visit
Onsite consent	X								
Pregnancy test	X	X	X	X	X	X	X	X	X
Medical history	X	X	X	X	X	X	X	X	X
Clinical assessments									
Ankle-brachial index	X		X		X		X		X
Audiometry	X		X		X		X		X
Cognition	X		X		X		X		X
Neuropsychiatric	X		X		X		X		X
Physical examination	X		X		X		X		X
Physical performance	X		X		X		X		X
Pulmonary function testing	X		X		X		X		X
Visual performance testing	X		X		X		X		X
Vitals	X	X	X	X	X	X	X	X	X
Biospecimen collection									
Blood	X	X	X	X	X	X	X	X	X
Saliva	X	X	X	X	X	X	X	X	X
Stool	X	X	X	X	X	X	X	X	X
Swabs (buccal, retroauricular, nares and oral microbiome)	X	X	X	X	X	X	X	X	X
Tears	X	X	X	X	X	X	X	X	X
Tissue					Throughout				
Urine	X	X	X	X	X	X	X	X	X
Imaging									
Coronary calcium scan	X								
Echocardiogram	X				X				
Stress echocardiogram	X				X				
Electrocardiogram	X				X				
Keratometry/corneal topography	X				X				X
Optical coherence tomography (OCT)	X				X				X
Posterioranterior (PA) and lateral chest X-ray	X								
Retinal photography	X				X				X
Sensors									
Study watch					Throughout				
Study hub					Throughout				
Sleep sensor					Throughout				
Mobile sensors					Throughout				
Self-reported data									
Self-reported data					Throughout				
Other									
Health records					Throughout				
Claims data					Throughout				

A broad range of health assessments are conducted in the deeply phenotyped cohort to capture potentially important markers of health and disease.

^a^For participants at elevated risk only. Assessments and biospecimen collection may be reduced over time depending on participant availability and compliance.

**Table 3:** Study vitals measured.

	Durham	Kannapolis	LA	Palo Alto	All sites
Systolic BP	125.4 ± 14.7	128.5 ± 16.5	121.9 ± 14.8	120.2 ± 16.1	123.2 ± 16
Diastolic BP	77.6 ± 10.9	78.8 ± 9.3	77.6 ± 9.5	72.6 ± 8.9	75.8 ± 9.9
Weight (kg)	85.7 ± 21.3	88.9 ± 22.8	77.7 ± 19.6	78.5 ± 20.4	81.9 ± 21.4
BMI	30.1 ± 7.2	31.2 ± 7.2	26.5 ± 6	27.2 ± 6.4	28.4 ± 6.9
Waist circumference (cm)	98.1 ± 17.2	97.1 ± 15.7	86.2 ± 15.8	90.9 ± 16.5	92.6 ± 16.9
Heart rate	67.1 ± 12	68.8 ± 12.1	67.8 ± 11.6	66.5 ± 11.1	67.4 ± 11.6
Respiratory rate	16.2 ± 2.4	16.4 ± 2.1	15.8 ± 1.6	14.9 ± 2.9	15.6 ± 2.5
Oxygen saturation	98.5 ± 1.6	98 ± 2.1	98.6 ± 1.6	98.7 ± 1.3	98.5 ± 1.6

A number of health vitals are collected in conjunction with other important health measures.

**Table 4:** Molecular measurements.

	*N*	Mean ± st dev	Median (IQR)
Lipid panel			
Total cholesterol (mg/dL)	2403	184 ± 40	183 (157, 209)
HDL (mg/dL)	2403	58.7 ± 19.0	56 (45, 69)
LDL (mg/dL)	2350	99.5 ± 33.7	97 (76, 120)
Triglycerides (mg/dL)	2403	135.1 ± 101.7	106 (76, 160)
hsCRP			
High sensitivity CRP (mg/L)	2393	2.9 ± 5.9	1.22 (0.59, 2.99)
ALAT (SGPT) (U/L)	2403	20.9 ± 14.2	17 (13, 24)
Albumin (g/dL)	2403	4.4 ± 0.3	4.4 (4.2, 4.6)
ASAT (SGOT) (U/L)	2403	21.4 ± 12.6	19 (16, 23)
Calcium (mg/dL)	2403	9.5 ± 0.4	9.5 (9.3, 9.7)
Chloride (mEq/L)	2403	102.9 ± 2.4	103 (102, 104)
Chemistry			
Creatinine (mg/dL)	2403	0.87 ± 0.32	0.84 (0.72, 0.97)
Magnesium (mEq/L)	2403	1.72 ± 0.15	1.7 (1.6, 1.8)
Potassium (mEq/L)	2403	4.3 ± 0.3	4.2 (4, 4.5)
Protein total serum (g/dL)	2403	7.0 ± 0.4	7 (6.8, 7.3)
Sodium (mEq/L)	2403	138.9 ± 2.1	139 (138, 140)
Uric acid (mg/dL)	2402	5.1 ± 1.3	5 (4.125, 5.9)
GFR			
MDRD (ML/M/1.73 M2)	2403	88.4 ± 20.4	87 (75, 100)
Hematocrit (%)	2392	43.3 ± 3.7	43.3 (40.7, 45.8)
Hemoglobin (g/dL)	2392	14.2 ± 1.3	14.2 (13.4, 15.1)
Hematology			
Neutrophils absolute (thousand/μl)	2372	3.93 ± 1.47	3.69 (2.91, 4.75)
Platelet count (per cubic mm)	2380	245487 ± 62543	238000 (203000, 281000)
White cell count (Thousand/μl)	2372	6.4 ± 1.9	6.15 (5.1, 7.4)
Hb A1c			
Hemoglobin A1c (%)	2404	5.7 ± 1.0	5.5 (5.2, 5.8)
TSH			
Thyroid stim hormone (MCIU/ML)	2398	1.81 ± 2.72	1.51 (1.06, 2.15)

A summary of molecular measurements made.

### Interactive and continuous assessments

Participant information is gathered from the web portal and the mobile app, which enable participants to provide regular updates on their health through structured and ad hoc questionnaires and surveys, existing and updated user interfaces, event reporting systems, and other mechanisms. Questionnaires cover a broad array of topics to better understand participants’ experience as being part of the study, history, environment, and other health-related information. Information that is collected includes: education, marital status, family size, household income data, personal and family health history, diet, physical activity, environmental factors, occupational exposures, functional capacity, mood (depression, anxiety, isolation), sense of well-being, behavioral characteristics across established domains (e.g., self-control, risk perception/risk taking, time discounting, rules/religion/habits, motivation/depression, general cognition), and sleep. The information collection will be adapted over time as the research goals of the initiative evolve.

Health monitoring is being implemented through approved and investigational sensor technology intended to maximize the proportion of a person’s time when measurements are made. During the initial course of the PBHS, participants are wearing a sensor device, which is built into a watch on their wrists and they use a sleep sensor to gather data as they rest. A separate network access point is available for uploading data from medical devices to the cloud. The time intervals for device use may vary throughout the study duration and device choices are tailored over time.

### Challenge studies

As the outcomes of the PBHS become available and hypotheses are generated, one method by which they are being tested is in the form of “challenge studies”. Challenge studies are intended to improve the measurement technology and to increase participant satisfaction and adherence to the protocol. For example, when a major modification of the algorithm to assess activity status using the study watch was made, participants were asked to record their activity on a frequent basis, yielding six million hours of labeled activity in a period of only 3 months in order to evaluate the algorithm. As the study progresses, the challenge studies will test possible interventions, such as changes in dietary or exercise parameters, behavior modifications, and other interventions likely to result in decreased disease risk or improved outcomes. This approach requires highly organized infrastructure for action and implementation through multiple systems, including social, educational and healthcare.

### Event ascertainment

Participants enrolled in the PBHS are encouraged to notify site personnel of changes in their health status or well-being and medical encounters. Biospecimens (e.g., blood samples) may be requested based on the occurrence of incident events while a participant is enrolled in the study with an initial focus on cancer and CVD events. Access to the EHR and claims data will support an understanding of medically significant follow-up events. Additional results from the workup (e.g., imaging results) may also be requested and incorporated into the participant dataset.

### Baseline expansion

The DPC is being expanded in a much broader, less detailed phenotyping effort using a collaborative set of networks. As the platform develops, it will be the core system for data collection and analysis for phenotyping specific populations entered into clinical care, or disease management or clinical research studies. For example, an organization has been formed to treat opioid addiction in which patients join a learning health system in which the integration of multiple data sources will be an integral part of the program^17^. Second, a network of health systems has been formed to better understand how to link participation in research with virtual and routine clinical elements^18^. Third, a consortium of pharmaceutical and device companies are identifying common needs for tools and methods, and beginning to use the platform for a variety of clinical studies. Finally, an initial collaboration with the American Heart Association is beginning to make the platform available to organizations representing patients with common, chronic and rare diseases and their families^19^. Thus, the deep phenotyping cohort is the foundation upon which a vast network of human studies is evolving.

### Return of results

**Fig. 2:**
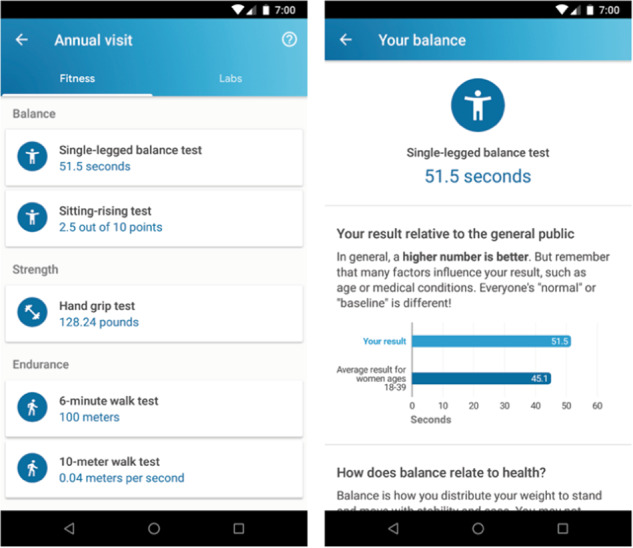
Results display. Results of physical performance testing are returned through the Project Baseline Mobile app. Results of each test are enhanced with contextual information including a description of the study procedure, normative data, and links to additional resources from both the lay and scientific communities.

The PBHS is committed to return results to participants. Return of results is important to inform participants of their own findings, potentially enhance motivation to remain in the study and improve retention and adherence. A Return of Results Committee was established to explore how results can be returned in a responsible and meaningful way without undue burden for participants, site teams, or clinicians caring for the participants in regular clinical care. The PBHS is committed to testing vehicles for the return of individual results^20^ that enhance the value of the participant’s data, such as coupling the return of research results with curated educational materials or with graphical displays to compare their individual results against aggregate results to help participants understand the findings in the appropriate context. Participants in the DPC receive personalized results from each of their study visits. Results of physical performance testing (Fig. 2) include measures of strength and balance obtained annually. Results are returned for each study visit along with normative data based on the particpant’s age and sex. Results are further contextualized by including links to lay and peer reviewed articles describing the testing and results in further detail. To date more than 70% of participatns in the DPC have viewed some of their results from the study.

If findings require immediate medical attention, the Return of Results Committee has developed protocols for participant notification based on acuity, actionability and the clinical judgement of site teams. Participants are encouraged to share their results with their clinician and to seek additional support if desired. Individual research results that may be returned include data from standard laboratory tests, clinical assessments, imaging, physical activity sensor data, survey data, and others.

Some data collected from the PBHS tests may be primarily of research value and not directly relevant to an individual’s health or clinically actionable. How to manage these data has been the source of considerable controversy^20^; empirical experience is needed to clarify the best approach. As the study progresses, more information may be required to inform if and how to return these results, while ensuring that the benefits of return of research results outweigh the unintended risks. For example, when a molecular laboratory test for which there are no well-established clinical benchmarks is included primarily for hypothesis generation, it could have unexpected consequences if individual results are misinterpreted by the participant, leading to unintended harm^21^. In the case of the UK Biobank return of imaging results from tests that were not indicated resulted in a higher number of invasive procedures for false positive findings (“incidentaloma”) relative to the number of useful clinical procedures result from return of results. Evaluation of the most effective methods of return of results will include external academic experts, the participants, their clinicians, and the research teams^22^.

Participants are able to elect whether to receive their genetic results. Participants who choose to receive genetic results receive a report from a gene panel and supportive counseling is provided should any discoveries occur for genes linked to a limited number of genetic conditions that are considered to be medically actionable^23^. Like with other results, participants are encouraged to share genetics results with their own clinicians, including potentially seeking additional genetic counseling.

As experience builds in returning individual research results, we will evaluate the benefits and risks of returning results, including participant understanding/satisfaction, any resulting change in participant behaviors, the impact on research teams and the clinicians caring for participants, and the timing, cost and potentially unintended consequences of returning results. These empirical data from the DPC will be reported for discussion by the scientific and patient communities, and will inform future policies for iterations of return of results procedures for the broader baseline cohort.

### Statistical approach and considerations

The initial cohort for the PBHS is expected to provide sufficient diversity to enable a variety of hypothesis-generating analyses. Identified trends will trigger follow-up studies with sufficient statistical power to test specific hypotheses. For the characterization of participant phenotypes, analyses will be based on the set of evaluable participants. In some cases, only subsets of participants or specified cohorts will be considered, or new cohorts will be created using the Baseline platform, either online or connected with clinicians, health systems, and advocacy groups.

A core concept of Baseline is that health and disease will be reclassified based on multidimensional analysis of systems biology. For example, simple issues such as depression status or diabetes diagnosis may impact pathways and organ systems not systematically measured jointly before. In essence, the DPC and the extended cohort could enable an extrapolation of the concept embodied by Patients Like Me: a much deeper and broader population^24^. Initial analyses will examine the relationships among various measures and disease states as well as the integration of multiple datasets. For study objectives related to biomarkers of health transitions and functional status, and for objectives comparing different phenotypic signals, a repeated-measures design assessing participants serially (e.g., within-participant analysis) will be employed to appropriately account for the dependency among measurements within the same participant. Machine learning methods will also be applied and are a focus for this study given the multidimensional nature of the data. Data captured through images and videos will be analyzed using convolutional neural networks or related methods^25,26^. For serially collected data with binary or continuous outcomes, recurrent neural networks or/and deep Poisson factor models will be implemented to relate trajectories of biomarkers to clinically relevant outcomes^27^.

A second core concept is “precision testing” in which it should be possible to predict whether a test under consideration is likely to yield useful additional information. For example, people with a normal 12 lead electrocardiogram may be less likely to have an abnormal echocardiogram and recent findings indicate that machine learning applied to routine ECG data will enhance its predictive value even further.

## Ethical considerations

Although this study aims to explore health, there is no expectation of direct health benefit to the participants. However, the information obtained will be used to advance knowledge that may be helpful to the participant or to the general population in the future. Overall, the potential risk is considered minimal and no greater than the risks associated with sample collection, radiographic studies, and the use of monitoring devices deployed in the study. Detection of unanticipated abnormal findings returned to participants may be of medical benefit but also could cause psychological distress or may also stimulate unnecessary testing or invasive follow-up procedures. Assessments of the views of study participants are collected periodically so that concerns of individuals can be addressed as the study proceeds. If additional, more interventional studies are planned, they will undergo specific protocol development and ethics committee review.

An independent Observational Study Monitoring Board (OSMB) is charged with monitoring participant safety in the DPC. As enrollment and initial sample ascertainment are now completed in the deeply phenotyped cohort, the OSMB is focused on emerging issues such as return of results. Since no experimental medical interventions are currently underway, the tasks are more subtle than the comparison of treatment groups that form a fundamental activity in trial data monitoring committees. In addition to ethical concerns, the practical issues in return of results have stimulated the formation of a special committee. An important area of concern is assurance of participant privacy and protection of individual rights for confidentiality of health information or health decisions. Access to sources of information that could be used to identify individual participants will be protected. Such harms arise from the disclosure of information, and privacy protections should prevent unplanned disclosure of individual information.

## Discussion

The PBHS aims to be an important step in a broader effort to develop a more comprehensive understanding of the nature of health and disease than previously possible. The unprecedented depth and volume of the multidimensional data generated by this study may lead to insights into the complex systems and interactions that influence states of health and disease, as well as the way we define these states. Although the concept of reclassification of health and disease has been accepted as a likely future direction, other than oncology, where molecular classification is superseding organ system classification in some cases^28^, the effort to understand disease at a more fundamental level has been limited by the tools available to measure and analyze multiple data dimensions. Over a longer period of time, the PBHS data repository could lead to improved real-time precision health interventions and virtual simulation models, such as a human health digital twin^5^.

The PBHS is being undertaken in the context of other major programs that aim to develop a deep understanding of human health^7–17^. In particular, the Terra data platform for the PBHS is being developed in concert with the All of Us Program, involving a collaboration with Vanderbilt University and the Broad Institute; this program plans to enroll at least 1 million participants. In the future, investigators will be able to access data from multiple existing studies as part of this platform, which is being developed as a comprehensive platform to enable organization, curation and analysis of previously disconnected streams of data.

The PBHS collects a broader and deeper array of data than most of the multiple, well-conceived epidemiological studies that have been conducted or have recently initiated enrollment^29–42^. While many studies are collecting deep and complex molecular information, few are measuring the combination of multidimensional features that includes, for example, genetic and molecular “–omics”, imaging, exercise testing, EHR and claims data, physiologic sensors, and wearables. Other key attributes of the study include increasing the frequency of health monitoring using wearable sensors and participant engagement using continuous approaches. When enough data are collected over time, the PBHS will provide a basis for interrogating human health using a systems approach for biomedicine.

Comparisons with the All of Us Study^43^ and the UK Biobank^44^ are particularly relevant. All of Us is a much larger study intended to enroll at least 1 million participants with plans to implement deep phenotyping over an extended period. The Terra platform is shared by the two studies and we expect that sharing insights or using one for validation of findings in the other will potentially be feasible. While the Baseline cohort from the expanded registry could be as large eventually, the specific measurements will be more dependent on the particular context of each study rather than the broad plan for All of Us. The UK Biobank has become a valuable public and private resource with deep phenotyping of 500,000 people in the UK between ages 40 and 69. It does not include the virtual expansion planned for Baseline, but it does have analogous depth of phenotyping with remarkable productivity already demonstrated. It has also developed a model for combining public access with protected data for time periods to enable development of intellectual property, a shared goal with the PBHS. Analogous similarities and differences could be depicted for most of the major epidemiological studies and cohorts underway.

The PBHS is intended to be a springboard for other similar approaches and can be expanded to include numerous disease areas and populations. While there are anticipated trends in disease rate and outcome based on known risk factors, this study could lead to a better understanding of how biomarkers and risk factors operate in systems biology. Likewise, this comprehensive evaluation could provide data that help reframe the way we describe particular disease conditions. For example, the molecular underpinnings of certain forms of cancer, metabolic, and cardiovascular diseases may have commonalities that go beyond traditional disease labels. The insights gained from this study will not be determined solely from the research groups involved and may not emerge quickly. The complexity of these comprehensive measurements likely will require data sharing and collaboration among multiple teams of biomedical researchers, data scientists, and clinical investigators to optimize the value of the analyses.

However, even with this potential for benefit, one must be mindful of other anticipated and unanticipated consequences of this level of human health evaluation. The balance of benefits and harms of such deep interrogation of biology, behavior and social interaction are not known, and efforts to intercede more quickly based on the type of more continuous monitoring conducted in this study could be complicated. Participants predisposed to early disease, who are receiving information about their genetic or “–omic” profile, must be protected from harm such as familial discord or personal psychological problems that might arise as a result. Just as considerable work is done to protect individuals from disease, work must also be carried out to ensure that these same individuals are protected from discrimination and other harms. In the United States, disclosure of genetic information by employers is protected by the Genetic Information Nondiscrimination Act of 2008^45^; however, this protection has limited case law supporting it, and is not designed to address non-genetic disease prediction. Many of these issues will need to be addressed prior to widespread adoption of surveillance approaches even if predictive strategies are discovered.

Furthermore, for this strategy to be widely implemented, it must be available to as many individuals as possible. However, just as this study will likely take many years to come to fruition, parallel efforts in health economics, regulation, and other related areas will be required, including the ethics of privacy, confidentiality and data sharing. New methods of monitoring health and detecting disease and its progression must be supported by health economic research that assures that their cost and labor intensity is justified by the operating characteristics of the predictive information.

## Limitations

The PBHS has inherent limitations. The decision was made for the study to measure more detail than most other studies in the initial phase, thereby limiting the number of participants in the DPC. This issue will be addressed both by data sharing strategies with ongoing studies and through the expansion of the study through the virtual, federated registry. Due to the length of follow-up and the substantial requirements of the deeply phenotyped participants because of the extensive measurements, participants’ adherence to the study protocol will not be complete. Every effort will be made, however, to help them do so, and the project’s commitment to engagement of participants will hopefully help minimize the issue. Additionally, while the study may have sufficient power to generate hypotheses and observe complex relationships among measures for standard assays, clinical assessments and outcomes, the sample size of the DPC will not be sufficient for some data analysis techniques. Furthermore, even though the overall study is recruiting a general population, it is also enriched for important disease risk areas relevant to the U.S. population, making the DPC part of the study limited to those areas and to the criteria specified by the study protocol. Similarly, an important challenge is the enrollment of the desired participant diversity with regard to education and income levels, given the limited number of enrollment sites. This will be addressed through the constellation of registries. Additionally, it should also be noted that the analyses, acquisitions, and known measures are only as good as the current technology permits and the measures and biomarkers of which the medical community is aware. Thus, future studies will evolve not only to improve technology, but build upon a body of prior knowledge of assessments known to be most relevant and informative within the scope of this study.

## Conclusions

The PBHS hopes to fill a significant gap in exploring the dynamic interplay of the biological, behavioral, environmental, and social systems and the impact of time that underlie health status. This study will be one of the most comprehensive collection, collation, and analysis of human health monitoring data in existence. These datasets comprise extensive measures, which will be built upon and mined for new insights, allowing for the generation of hypotheses about how different systems are interdigitated in health and disease. The study is building a platform that will connect a federation of virtual and physical registries and data will be available to the scientific community to encourage learning across studies and platforms. The overall intention is to discover signals that will lead to subsequent confirmatory studies by setting the stage for a transition in the approach to understanding, predicting and detecting human disease from limited analysis of several types of data to the use of multidimensional analysis.

## Supplementary information

Supplementary Information

## Data Availability

Data sharing not applicable to this article as no datasets were analysed during the current study. However, future data access will be possible for qualified investigators pending Project Baseline committee approval.
