# Supplementary material for: The Project Baseline Health Study: a step towards a broader mission to map human health
Source: NPJ Digit Med. 2020 Jun 5;3:84. doi: 10.1038/s41746-020-0290-y (PMC7275087; doi:10.1038/s41746-020-0290-y)
Supplement: Supplementary file 1 — Supplementary Information [file 41746_2020_290_MOESM1_ESM.pdf]

Supplementary Materials

Study Design

Stratified Enrollment by Risk

Baseline Registry participants will be selected to obtain a study population enriched for participants with an elevated risk of CVD, lung cancer, and breast/ovarian cancers.

Elevated Risk for Cardiovascular Disease

Within each age strata, 60% of the total enrolled population should meet all of the following criteria for elevated CVD risk:

- No prior or current atherosclerotic cardiovascular disease (ASCVD); including prior myocardial infarction, stroke, surgery, amputations, significant blockages, or stent placed for blockages in the carotids, coronaries, or peripherals as reported by the participant.
- No current cancer as reported by the participant
- Upper 60th percentile of risk scores for CVD risk relative to the distribution of risks for the same age and sex observed in the interview sample population of the 2011-2012 NHANES survey. Participants risks and NHANES survey [1] risks are calculated using published risk scores as follows:
    - Framingham Risk Score for 30-year risk of hard CVD events for those ages 18-39 years [2]
    - 2013 American College of Cardiology/American Heart Association (ACC/AHA) ASCVD risk estimation equation for 10-year risk for hard CVD events for those ages 40+ years [3]

Elevated Risk for Lung Cancer

Within each age strata, 60% of the total enrolled population should meet all of the following criteria for elevated risk of lung cancer:

- No prior or current cancer as reported by the participant (except non-melanoma skin cancer)
- Must meet at least one of these criteria for elevated lung cancer risk:
    - Upper 60th percentile of lung cancer risk [4] relative to the distribution of cancer risks for the same age and sex observed in the subpopulation of smokers in the 2010 NHIS Cancer Control Supplement (CCS) [5]
    - Current cigarette smoking with cumulative ≥ 30 pack year history
    - Previous cumulative cigarette smoking history ≥ 30 pack year and quit < 15 years prior to enrollment

Elevated Risk for Breast/Ovarian Cancers

Within each age strata, 60% of the total enrolled female population should meet all of the following criteria for elevated risk of breast/ovarian cancers:

- No prior or current cancer as reported by the participant (except non-melanoma skin cancer)
- No prophylactic cancer surgery (bilateral mastectomy, risk-reducing salpingo-oophorectomy) as reported by the participant
- Must meet at least one of these criteria for elevated breast/ovarian cancer risk:

- Upper 60th percentile of breast/ovarian cancers risk [6] relative to the distribution of cancer risk for the same age and sex observed in the 2010 NHIS CCS
- Known carrier of a genetic mutation associated with breast/ovarian cancers (BRCA1, BRCA2)

Participants with an elevated breast/ovarian cancer risk will be female, reflecting conventional risk model's [6] definition of breast/ovarian cancer risk only among females.

Data Access

The Baseline project data will be available to qualified researchers for exploratory analysis in the future. Qualified external researchers may apply to use the data and samples from the Baseline project through applications reviewed by the Proposal Review and Publications Committee. There will be appropriate safeguards to protect individual participant privacy.

Ethical Considerations

Consent procedures and documentation will comply with IRB. Signed informed consent and HIPAA authorization will be obtained from each participant in the Project Baseline study or from the participant's Legally Authorized Representative (LAR). Verily will keep health information on secure Google third party systems with many layers of protection.

Study Committee Members:

Members of study governance Committees including the Scientific Executive Committee and Publications Committee, Return of Results Committee, Observational Study Monitoring Board, and Participant Representatives Participating in Engagement Committee are as follows.

G. Bernard, R. Bigelow, W. Bowman-Zatzkin, R. Califf, A. Conrad, A. C. Fan, S. S. Gambhir, R. Green, A. P. Heagerty, A. Hernandez, S. Krug, J. D. Lantos, K. W. Mahaffey, S. McLaurin, J. Mega, K. B. Orrico, C. J. Pepine, J. Perimutter, M. Pigone, R. L. Schilsky, S. Shah, S. Torres, C. Wong, L. Wruck.

Supplementary References

1. NHANES Questionnaires, Datasets, and Related Documentation. Available at: https://wwwn.cdc.gov/nchs/nhanes/ContinuousNhanes/Default.aspx?BeginYear=2011. (Accessed: 16th January 2019)
2. Pencina, M. J., D'Agostino, R. B., Larson, M. G., Massaro, J. M. & Vasan, R. S. Predicting the 30-year risk of cardiovascular disease: The framingham heart study. *Circulation* (2009). doi:10.1161/CIRCULATIONAHA.108.816694
3. Goff, D. C. *et al.* 2013 ACC/AHA guideline on the assessment of cardiovascular risk: A report of the American college of cardiology/American heart association task force on practice guidelines. *Journal of the American College of Cardiology* (2014). doi:10.1016/j.jacc.2013.11.005
4. Bach, P. B. *et al.* Variations in Lung cancer risk among smokers. *J. Natl. Cancer Inst.* (2003). doi:10.1093/jnci/95.6.470
5. National Health Interview Survey (NHIS) Cancer Control Supplement (CCS). Available at: https://healthcaredelivery.cancer.gov/nhis/. (Accessed: 16th January 2019)
6. Costantino, J. P. *et al.* Validation studies for models projecting the risk of invasive and total breast cancer incidence. *J. Natl. Cancer Inst.* (1999). doi:10.1093/jnci/91.18.1541

Supplementary Figures

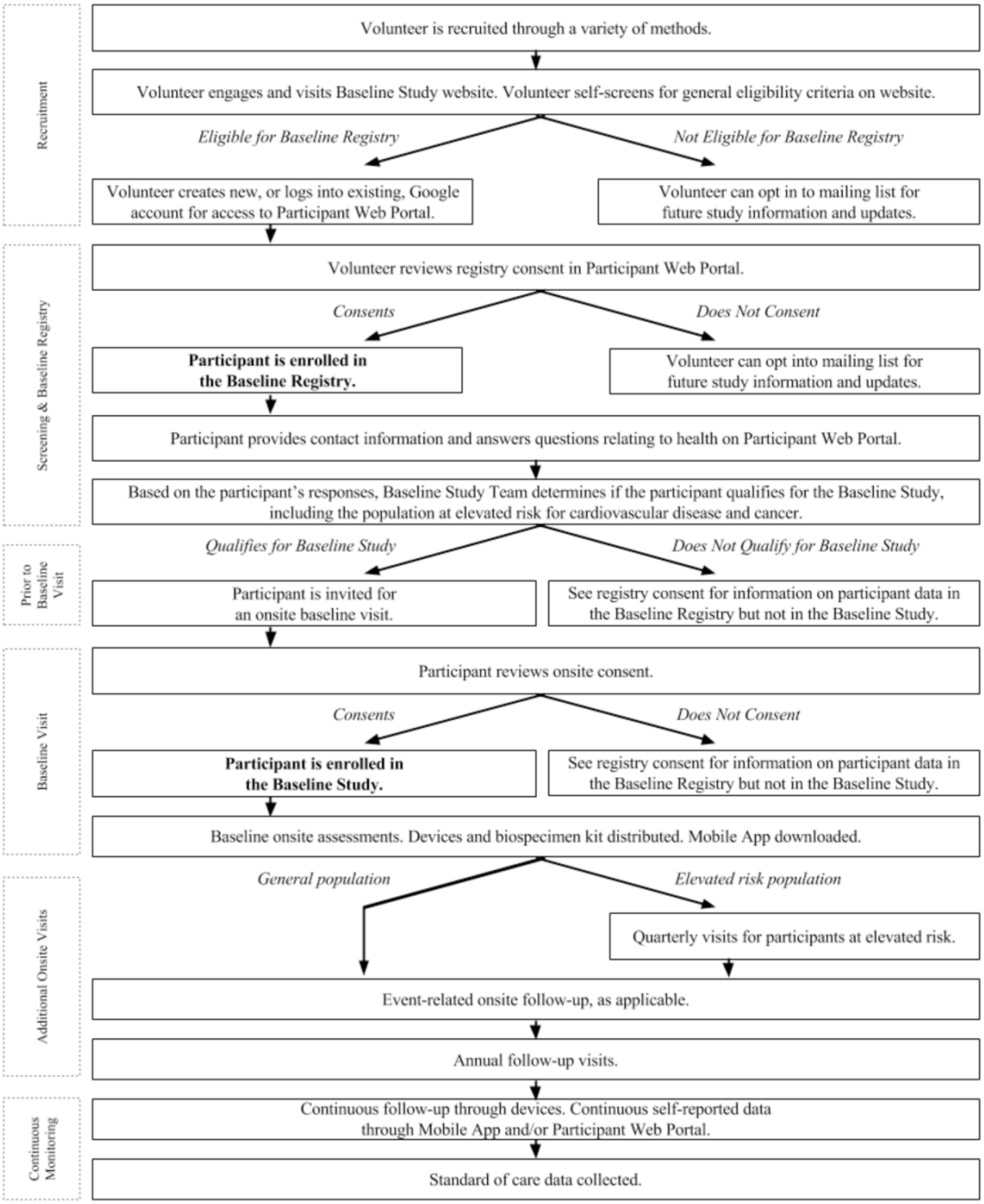

Supplementary Figure 1. Schematic of Study Design

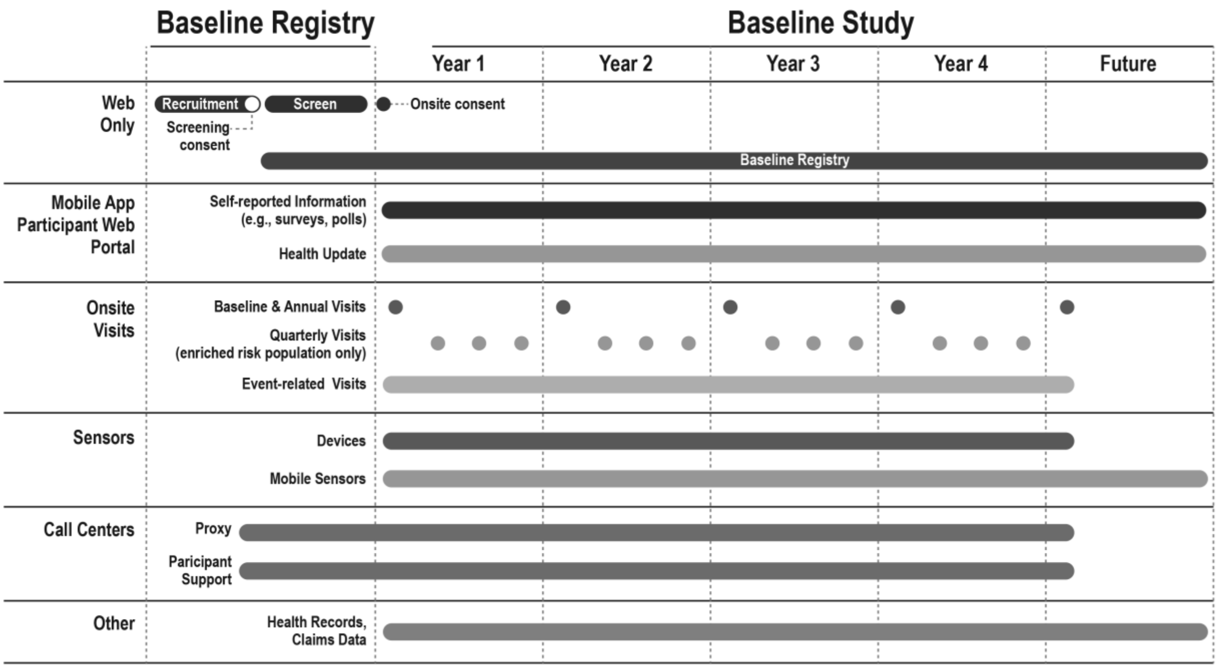

Supplementary Figure 2. Schematic of Study Schedule
